# Cross-sectional mediation analysis of systemic inflammation in the association between serum uric acid and diabetic kidney disease: Evidence from NHANES 1999–2018

**DOI:** 10.1016/j.metop.2025.100426

**Published:** 2025-12-02

**Authors:** Jiaying Wang, Weijing Liu, Jiaoyan Li, Mengxiao Li, Heyan Feng, Shangfei Liu, Yanzhe Cheng, Wei Li

**Affiliations:** a The Third Affiliated Hospital of Beijing University of Chinese Medicine, Beijing, China; b Dongzhimen Hospital of Beijing University of Chinese Medicine, Beijing, China; c Beijing Integrated Traditional Chinese and Western Medicine Consultation Center for Difficult Kidney Diseases, Beijing, China

## Abstract

**Objective:**

To investigate whether systemic inflammation, quantified by the Aggregate Index of Systemic Inflammation (AISI), mediates the association between uric acid (UA) and diabetic kidney disease (DKD).

**Study design:**

We analyzed data from 1716 adults with diabetes in NHANES 1999–2018. We used regression to assess UA-AISI-DKD associations and mediation analysis to quantify AISI's indirect effect. A random forest model, interpreted via SHAP, predicted DKD risk.

**Results:**

Each 1 mg/dL increase in UA was associated with a 14 % higher DKD risk (adjusted OR = 1.14, 95 % CI: 1.04–1.26). UA was positively associated with AISI (β = 0.0356, p = 0.0058), which in turn predicted DKD (OR = 1.25 per SD increase in ln-AISI, 95 % CI: 1.10–1.42). AISI partially mediates (10.94 %) the association between UA and DKD, indicating that systemic inflammation is one of several pathways linking hyperuricemia to renal injury. The random forest model performed best, with SHAP highlighting AISI as a key positive predictor.

**Conclusion:**

Systemic inflammation, as measured by AISI, partially mediates the cross-sectional association between serum uric acid and diabetic kidney disease, supporting inflammation as one of several contributing pathways. The predictive performance of models incorporating AISI remains modest and does not outperform conventional clinical risk scores.

## 1 Introduction

Diabetic kidney disease (DKD) is one of the leading causes of chronic kidney disease (CKD) and end-stage renal disease (ESRD) worldwide, imposing a substantial burden on public health and healthcare systems [1,2].With the incidence of DKD showing a trend toward younger populations, its prevalence is projected to increase by nearly 50 % over the next 25 years, from 537 million to 783 million cases [3].Among its multiple risk factors, hyperuricemia (HUA) has increasingly been recognized as an important and modifiable contributor to the onset and progression of DKD.Epidemiological and mechanistic studies have demonstrated that elevated uric acid (UA) not only induces kidney injury through urate crystal–dependent pathways, but also exacerbates renal pathology via non-crystalline mechanisms, such as activation of the renin–angiotensin–aldosterone system (RAAS), endothelial dysfunction, and oxidative stress [[4], [5], [6]]. Moreover, hyperuricemia intensifies diabetes-related complications in the heart, kidneys, and retina [7,8], ultimately heightening the risk of mortality [9].

The Expert Consensus on Pharmacological Uric Acid–Lowering Therapy for High-Risk Hyperuricemia (2025 Edition) [10] emphasizes that persistent elevation of serum uric acid, particularly in high-risk populations such as patients with diabetes, is closely associated with accelerated renal function decline. It further highlights systemic inflammation as the central intermediate link connecting hyperuricemia to terminal target-organ damage. The consensus recommends regular evaluation of systemic inflammatory status in high-risk HUA patients to enable more precise risk monitoring. This perspective reflects a paradigm shift from focusing solely on uric acid reduction to simultaneously targeting uric acid–driven inflammatory pathways. In terms of treatment, the consensus notes that xanthine oxidase inhibitors (e.g., allopurinol, febuxostat) not only suppress uric acid synthesis but also exert anti-inflammatory effects. These agents have been shown to inhibit reactive oxygen species production, attenuate NLRP3 inflammasome activation, and downregulate proinflammatory cytokines such as interleukin-1β (IL-1β) and interleukin-6 (IL-6), thereby potentially interrupting the “UA–inflammation–renal injury” cascade [11,12]. Such anti-inflammatory properties provide strong rationale for incorporating inflammation monitoring into the management of hyperuricemia in patients with diabetic nephropathy [13].

The aggregate index of systemic inflammation (AISI) is an emerging biomarker that integrates neutrophil, lymphocyte, monocyte, and platelet counts, calculated as AISI = (neutrophils × platelets × monocytes)/lymphocytes. Compared with single inflammatory markers such as C-reactive protein (CRP) or interleukin-6 (IL-6), AISI provides a more comprehensive reflection of systemic inflammatory status. To date, it has demonstrated remarkable prognostic value in diverse conditions, including cancer [14], infectious diseases [15], acute coronary syndrome [16], and hypertension [17]. Moreover, population-based analyses using the NHANES dataset have shown that elevated AISI levels are significantly associated with increased risks of chronic kidney disease and reduced estimated glomerular filtration rate [18].

Building upon the above theoretical and clinical evidence, we propose the core hypothesis that UA may contribute to the onset and progression of DKD primarily by triggering systemic inflammation. To test this hypothesis, we employed the nationally representative NHANES dataset to investigate the mediating role of the aggregate AISI in the relationship between UA and DKD. This study not only provides epidemiological support for the metabolism–inflammation–organ damage paradigm, but also offers a theoretical foundation for future precision prevention strategies targeting inflammation in diabetic populations.

## 2 Methods

### 2.1 Study design and population

Data for this study were obtained from the National Health and Nutrition Examination Survey (NHANES), a publicly available database covering the years 1999–2018. NHANES employs a multistage, stratified, probability sampling design to collect nationally representative data on the health and nutritional status of the U.S. population, with each survey cycle conducted biennially. To increase statistical power, we combined 10 consecutive cycles (1999–2018) and adjusted sampling weights according to the official NHANES analytical guidelines [17,19]. For participants from 1999 to 2002, the final weight was calculated as WTMEC4YR/2. For participants from 2003 to 2018, the final weight was calculated as WTMEC2YR/8. The stratification variable was SDMVSTRA, and the clustering variable was SDMVPSU. All NHANES data are de-identified and publicly accessible. The survey protocol was approved by the National Center for Health Statistics (NCHS) Ethics Review Board, and informed consent was obtained from all participants [18,20]. As this was a cross-sectional analysis based entirely on anonymized data, no additional ethical approval was required. Throughout the study, we adhered strictly to NHANES data use policies and ethical standards.

**Fig. 1:**
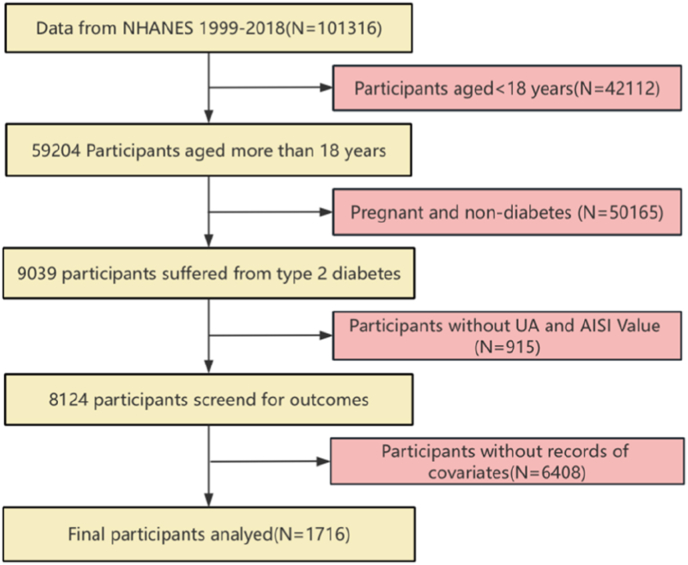
Flowchart of study participants.

For this prospective cohort analysis, we screened and evaluated NHANES data spanning 10 biennial cycles from 1999 to 2018. To ensure the integrity and reliability of the results, we applied the following exclusion criteria: (1) individuals younger than 18 years (N = 42,112); (2) pregnant participants and those without diabetes (N = 50,165); (3) individuals with insufficient data to calculate AISI or missing serum uric acid values (N = 915); and (4) individuals lacking records of essential covariates (N = 6408). After applying these criteria, a total of 1716 participants were included in the final analysis (Fig. 1).

### 2.2 Assessment of AISI

The AISI was calculated using the formula [21]:

$$\text{AISI}=\left(\text{neutrophil}\;\text{count}\right)\times \left(\text{monocyte}\;\text{count}\right)\times \left(\text{platelet}\;\text{count}\right)/\left(\text{lymphocyte}\;\text{count}\right).$$

In our study, AISI was regarded as a continuous exposure variable, and all participants were stratified into tertiles based on AISI values for subsequent analysis.

### 2.3 Definition of DKD, low-eGFR, and albuminuria

Self-reported diabetes, the use of insulin or other diabetes drugs, or specific criteria based on fasting glucose (mmol/l) ≥ 7.0 or glycosylated hemoglobin A1c (HbA1c) (%) > 6.5 were all required for the diagnosis of diabetes. DKD was diagnosed with the low estimated glomerular filtration rate (eGFR) (<60 mL/min/1.73 m2) or albuminuria (urinary albumin-to-creatinine ratio (ACR) ≥30 mg/g) in T2DM patients [22]. The eGFR was calculated using the Chronic Kidney Disease Epidemiology Collaboration (CKD-EPI) equation for standardized creatinine [23].The main outcome variables in this study were albuminuria, low-eGFR, and DKD.

### 2.4 Covariates

The following covariates were collected for analysis: sex, age, race/ethnicity, educational level, marital status, family poverty-income ratio (PIR), smoking status, alcohol consumption, physical activity, hypertension, hyperlipidemia, cardiovascular disease, diabetes, body mass index (BMI), HbA1c, fasting plasma glucose (FPG), serum creatinine, blood urea nitrogen (BUN), hemoglobin, lymphocyte count, neutrophil count, monocyte count, platelet count,ACR and eGFR.

BMI was calculated as weight (kg) divided by height squared (m^2^). Smoking status was categorized as never, former, or current based on responses to the questions: “Have you smoked at least 100 cigarettes in your life?” (SMQ020) and “Do you now smoke cigarettes?” (SMQ040). Alcohol consumption was defined using the questions “Have you had at least 12 drinks in any one year?” (ALQ101 or ALQ111). Participants were grouped into two categories: >12 drinks/year and <12 drinks/year, with one drink equivalent to 12 ounces of beer, 5 ounces of wine, or 1.5 ounces of liquor. eGFR was calculated using the Chronic Kidney Disease Epidemiology Collaboration (CKD-EPI) equation [24].Self-reported questionnaires were used to ascertain the presence of hypertension (BPD035), hyperlipidemia (BPD035), cardiovascular disease (BPD035), and diabetes (BPD035). Specifically, hypertension was defined by a physician's diagnosis (BPQ020), use of antihypertensive medication (BPQ040A), or blood pressure measurements (systolic BPXSY1, diastolic BPXDI1). Hyperlipidemia was determined using laboratory measurements (total cholesterol LBXTC, triglycerides LBXTR, low-density lipoprotein cholesterol LBDLDL, high-density lipoprotein cholesterol LBDHDD). Cardiovascular disease was identified based on questionnaire items including heart failure (MCQ160B), coronary heart disease (MCQ160C), and heart attack (MCQ160E).

$$\text{eGFRCKD}-\text{EPI}\left(\text{mL}/\min/1.73m^{2}\right)=141\times \min\left(\text{Scr}/\kappa ,1\right)ɑ\times \max\left(\text{Scr}/\kappa ,1\right)-1.209\times 0.993\text{Age}\times 1.018\left[\text{if}\;\text{female}\right]\times 1.159\left[\text{if}\;\text{black}\right]$$

where Scr is serum creatinine,κis 0.7 for women and 0.9 for men,αis −0.329 for women and −0.411 for men, min indicates the minimum value of Scr/κor 1, and max indicates the maximum value of Scr/κ or 1.

UACR(mg/g)=MAU(mg/L)/Ucr(g/L)

## 3 Machine learning feature preprocessing and selection

The SMOTE method was applied to address the imbalance between minority and majority groups. This technique calculates distances between minority class samples, identifies each sample's k-nearest neighbors, and generates synthetic samples along randomly selected directions between these neighbors. Consequently, the number of minority samples increased, mitigating class imbalance. To avoid disproportionate effects of feature values during training, all features were standardized.

## 4 Statistical analysis

All statistical analyses were performed using Python software (version 3.9). Continuous variables were expressed as medians with interquartile ranges (IQRs), and categorical variables were summarized as counts with percentages. Between-group comparisons were conducted using the Kruskal–Wallis test for continuous variables and the chi-square test for categorical variables. A two-sided p value < 0.05 was considered statistically significant.

Multivariable logistic regression models were applied to estimate the association between UA levels and the prevalence of DKD. Results were reported as odds ratios (ORs) with 95 % confidence intervals (CIs). Model 1 was unadjusted. Model 2 was adjusted for demographic and socioeconomic variables, including sex, age, race/ethnicity, education, family income, and marital status. Based on Model 2, Model 3 was further adjusted for lifestyle and clinical factors, including smoking status, alcohol consumption, physical activity,BMI, HbA1c, insulin use, oral hypoglycemic drug use, diabetes duration, fasting plasma glucose, serum creatinine, BUN, as well as histories of hyperlipidemia, hypertension, and cardiovascular disease. The ORs derived from the three models are presented in Table 1.

**Table 1:** Characteristics of the study population with uric acid levels.

Characteristics	Overall	UA			P-value
	(N = 1716)	Low(N = 417)	Medium(N = 571)	High(N = 728)	
Gender					<0.001
Female	802 (46.7 %)	258 (61.9 %)	274 (48.0 %)	270 (37.1 %)	
Male	914 (53.3 %)	159 (38.1 %)	297 (52.0 %)	458 (62.9 %)	
Age,years	62.02 ± 12.77	59.36 ± 13.17	62.15 ± 12.65	63.44 ± 12.40	<0.001
Race					<0.001
Mexican American	314 (18.3 %)	96 (23.0 %)	118 (20.7 %)	100 (13.7 %)	
Non-Hispanic Black	416 (24.2 %)	87 (20.9 %)	138 (24.2 %)	191 (26.2 %)	
Non-Hispanic White	650 (37.9 %)	141 (33.8 %)	200 (35.0 %)	309 (42.4 %)	
Other Hispanic	183 (10.7 %)	61 (14.6 %)	61 (10.7 %)	61 (8.4 %)	
Other Race	153 (8.9 %)	32 (7.7 %)	54 (9.5 %)	67 (9.2 %)	
Education					0.0281
Above high school	750 (43.7 %)	161 (38.6 %)	243 (42.6 %)	346 (47.5 %)	
Below high school	571 (33.3 %)	159 (38.1 %)	195 (34.2 %)	217 (29.8 %)	
High school	395 (23.0 %)	97 (23.3 %)	133 (23.3 %)	165 (22.7 %)	
Marital					0.031
Coupled	1035 (60.3 %)	229 (54.9 %)	350 (61.3 %)	456 (62.6 %)	
Single/Separated	681 (39.7 %)	188 (45.1 %)	221 (38.7 %)	272 (37.4 %)	
PIR					0.0286
1.30–3.49	733 (42.7 %)	182 (43.6 %)	229 (40.1 %)	322 (44.2 %)	
<1.29	556 (32.4 %)	152 (36.5 %)	182 (31.9 %)	222 (30.5 %)	
>3.50	427 (24.9 %)	83 (19.9 %)	160 (28.0 %)	184 (25.3 %)	
Smoking					0.0085
Current	264 (15.4 %)	77 (18.5 %)	82 (14.4 %)	105 (14.4 %)	
Former	573 (33.4 %)	116 (27.8 %)	184 (32.2 %)	273 (37.5 %)	
Never	879 (51.2 %)	224 (53.7 %)	305 (53.4 %)	350 (48.1 %)	
Drinking					0.0069
<12 drinks/year	542 (31.6 %)	146 (35.0 %)	196 (34.3 %)	200 (27.5 %)	
>12 drinks/year	1174 (68.4 %)	271 (65.0 %)	375 (65.7 %)	528 (72.5 %)	
Physical exercise					0.7913
Active	205 (11.9 %)	53 (12.7 %)	76 (13.3 %)	76 (10.4 %)	
Highly active	513 (29.9 %)	126 (30.2 %)	170 (29.8 %)	217 (29.8 %)	
Inactive	684 (39.9 %)	165 (39.6 %)	222 (38.9 %)	297 (40.8 %)	
Insufficient	314 (18.3 %)	73 (17.5 %)	103 (18.0 %)	138 (19.0 %)	
BMI					<0.001
Obese (>30)	975 (56.8 %)	201 (48.2 %)	300 (52.5 %)	474 (65.1 %)	
Overweight (25_30)	515 (30.0 %)	136 (32.6 %)	185 (32.4 %)	194 (26.6 %)	
Healthy weight (18.5_25)	219 (12.8 %)	75 (18.0 %)	86 (15.1 %)	58 (8.0 %)	
Underweight (<18.5)	7 (0.4 %)	5 (1.2 %)	0 (0.0 %)	2 (0.3 %)	
HTN					<0.001
Yes	1419 (82.7 %)	314 (75.3 %)	464 (81.3 %)	641 (88.0 %)	
No	297 (17.3 %)	103 (24.7 %)	107 (18.7 %)	87 (12.0 %)	
CVD					<0.001
Yes	358 (20.9 %)	67 (16.1 %)	98 (17.2 %)	193 (26.5 %)	
No	1358 (79.1 %)	350 (83.9 %)	473 (82.8 %)	535 (73.5 %)	
Insulin_use					0.0066
Yes	481 (28.0 %)	135 (32.4 %)	140 (24.5 %)	206 (28.3 %)	
No	1235 (72.0 %)	282 (67.6 %)	431 (75.5 %)	522 (71.7 %)	
Oral hypoglycemic use					0.5292
Yes	1259 (73.4 %)	291 (69.8 %)	423 (74.1 %)	545 (74.9 %)	
No	457 (26.6 %)	126 (30.2 %)	148 (25.9 %)	183 (25.1 %)	
DM duration years	11.81 ± 10.98	11.57 ± 10.02	11.52 ± 10.74	12.17 ± 11.68	0.7585
DKD					<0.001
Yes	713 (41.6 %)	117 (28.1 %)	196 (34.3 %)	400 (54.9 %)	
No	1003 (58.4 %)	300 (71.9 %)	375 (65.7 %)	328 (45.1 %)	
HLD					<0.001
Yes	629 (36.7 %)	128 (30.7 %)	193 (33.8 %)	308 (42.3 %)	
No	1087 (63.3 %)	289 (69.3 %)	378 (66.2 %)	420 (57.7 %)	
BMI	32.15 ± 7.29	30.57 ± 6.79	31.46 ± 6.81	33.59 ± 7.67	<0.001
Scr,umol/L	88.91 ± 57.40	71.90 ± 42.19	80.95 ± 40.93	104.90 ± 70.48	<0.001
Lymphocyte,10^9^/L	2.04 ± 0.91	2.00 ± 0.71	2.09 ± 1.19	2.02 ± 0.76	0.5149
Neutrophils,10^9^/L	4.36 ± 1.65	4.26 ± 1.56	4.24 ± 1.59	4.52 ± 1.74	0.0035
Monocyte,10^9^/L	0.56 ± 0.19	0.52 0.18	0.54 ± 0.18	0.59 ± 0.21	<0.001
Platelet count,10^9^/L	237.02 ± 72.00	246.52 ± 76.62	233.91 ± 67.12	234.02 ± 72.59	0.005
WBC,10^9^/L	7.23 ± 2.13	7.03 ± 1.96	7.14 ± 2.24	7.42 ± 2.13	<0.001
HGB, g/dL	13.83 ± 1.64	13.80 ± 1.45	13.87 ± 1.64	13.81 ± 1.73	0.7302
HbA1c,%	7.44 ± 1.78	8.00 ± 2.12	7.38 ± 1.73	7.16 ± 1.51	<0.001
FPG,mmol/L	8.78 ± 3.50	9.87 ± 4.24	8.61 ± 3.20	8.28 ± 3.10	<0.001
TC,mg/dL	178.04 ± 42.66	181.06 ± 43.76	179.41 ± 40.30	175.23 ± 43.70	0.0093
HDL-C, mg/dL	49.54 ± 13.90	52.38 ± 14.52	50.31 ± 13.76	47.31 ± 13.30	<0.001
LDL-C,mg/dL	100.85 ± 37.06	102.11 ± 38.97	102.33 ± 35.08	98.96 ± 37.41	0.0632
TG,mg/dL	138.25 ± 70.14	132.90 ± 69.79	133.90 ± 67.89	144.72 ± 71.63	0.0032
AISI	330.20 ± 295.98	310.98 ± 254.37	303.44 ± 276.95	362.20 ± 328.17	<0.001
eGFR, mL/min/1.73 m^2^	81.94 ± 25.46	93.60 ± 21.54	85.89 ± 22.81	72.17 ± 25.88	<0.001
UACR,mg/g	178.83 ± 778.57	107.71 ± 691.14	118.34 ± 667.99	267.02 ± 890.62	0.0084
BUN,mmol/L	2.15 ± 1.07	1.72 ± 0.64	2.01 ± 0.83	2.51 ± 1.30	<0.001

In addition, multivariable logistic regression analyses were performed to evaluate the associations of AISI with both UA levels and the prevalence of DKD. As an integrated index, AISI incorporates three major pro-inflammatory components—neutrophils, monocytes, and platelets—in the numerator, while using lymphocytes in the denominator to reflect the dynamic balance between “pro-inflammatory drive” and “immune regulatory capacity.”

For the mediation analysis, we evaluated the mediating effects of inflammatory markers (AISI, neutrophils, monocytes, platelets, and lymphocytes) on the relationship between UA and DKD. All models were adjusted for sex, age, race/ethnicity, educational level, family income, marital status, smoking status, alcohol consumption, physical activity,BMI, HbA1c, insulin use, oral hypoglycemic drug use, diabetes duration, fasting plasma glucose, serum creatinine, BUN, hyperlipidemia, hypertension, and cardiovascular disease. Among these, HbA1c, serum creatinine, and BUN were considered covariates directly related to DKD (as AISI may influence DKD indirectly through these factors). Therefore, they were not included when analyzing the association between AISI and UA.The presence of a mediation effect was defined as fulfilling all of the following criteria: (1) a statistically significant indirect effect, (2) a significant total effect, and (3) a positive proportion mediated.

The dataset was divided into training and validation sets at a 7:3 ratio to minimize overfitting. Six machine learning models were developed using the MLR3 framework: Random Forest, LightGBM, K-KNN, Naive Bayes, SVM, and XGBoost. Random Forest aggregates results from multiple decision trees, providing strong generalization, effective multiclass handling, and resistance to overfitting. LightGBM, based on gradient-boosted decision trees, improves training speed and efficiency while minimizing memory consumption. It achieves high accuracy, supports parallel learning, and is suitable for classification and regression tasks. K-KNN classifies or regresses based on the nearest data points. Its advantages include simplicity, no training stage, and effectiveness for small datasets and nonlinear problems. Naive Bayes, derived from Bayes’ theorem and the assumption of feature independence, offers high computational efficiency and robustness to incomplete data. SVM identifies the optimal separating hyperplane for classification, effectively handling high-dimensional data and distinguishing complex decision boundaries. XGBoost, an ensemble method based on gradient boosting, enhances predictions by combining weak learners. It provides fast optimization, tolerance to missing values, and strong performance with large-scale datasets.

Benchmarking was performed to systematically compare model performance using standardized datasets and consistent evaluation metrics. Six indicators were applied: Accuracy, F-Beta, ROC AUC, Sensitivity, Specificity, and PR AUC. ROC AUC was regarded as the primary metric, while the others served as secondary indicators. Ten-fold cross-validation was used to improve stability. Differences among models were evaluated using ANOVA and the Kruskal–Wallis H test. All machine-learning comparisons were performed with 10-fold cross-validation and that differences between models were tested using one-way ANOVA with post-hoc Bonferroni correction.

SHAP (SHapley Additive exPlanations) and LIME (Local Interpretable Model-agnostic Explanations) were employed to interpret feature contributions in the best-performing model. SHAP, based on the Shapley value from game theory, quantifies the contribution of each feature by considering all possible feature combinations. It supports interpretation of complex models and feature importance analysis. LIME, in contrast, approximates complex model behavior locally with simple interpretable models such as linear regression, thereby providing explanations for individual predictions.

## 5 Results

### 5.1 Participants characteristics

Table 1 presents the baseline characteristics of participants across triquels of serum UA. In the overall population, the mean UA level was 5.80 ± 1.54 mg/dL, ranging from 3.98 ± 0.55 mg/dL in Q1, 5.32 ± 0.35 mg/dL in Q2, to 7.23 ± 1.08 mg/dL in Q3. We observed that with increasing UA levels, the proportion of male participants markedly increased, the elderly were more represented, and the mean age rose by approximately 4 years. Obesity emerged as a strong risk factor, accounting for 65.1 % in the high-UA group. Kidney function showed notable deterioration, with eGFR declining by 22.9 % (from 93.60 to 72.17 mL/min/1.73 m^2^), serum creatinine increasing by more than 45.9 % (71.90 → 104.9 μmol/L), and UACR rising by over 147.9 % (107.71 → 267.02 mg/g). Neutrophil and monocyte counts also increased significantly. Moreover, the prevalence of hypertension, cardiovascular disease, and hyperlipidemia was higher in the high-UA group. Interestingly, despite a higher burden of kidney disease, the high-UA group exhibited lower blood glucose levels, which may be attributable to impaired renal function—where reduced eGFR leads to falsely lowered HbA1c values due to shortened red blood cell lifespan—or to potential treatment-related bias.

### 5.2 Associations of uric acid levels with DKD

Among the 1716 participants included in this study, a total of 713 were diagnosed with DKD, yielding a prevalence of 41.6 %. Table 2 summarizes the associations between UA levels and DKD prevalence. In Model 1 (unadjusted), participants in the highest UA group had a 3.13-fold increased risk of DKD compared with those in the lowest group. After adjusting for all potential confounders in Model 3, DKD prevalence continued to rise with increasing UA levels, demonstrating a significant positive association. Specifically, each 1 mg/dL increase in UA was associated with a 14 % higher risk of DKD, while participants in the highest UA group showed a 43 % higher risk (OR = 1.43, 95 % CI = 1.02–2.00).

**Table 2:** The associations of uric acid levels with DKD.

DKD	OR (95 % CI)		
	Model1	Model2	Model3
Continuous uric acid			
uric acids	1.47 (1.37,1.57)	1.46 (1.35, 1.57)	1.14(1.04, 1.26)
Uric acid categories			
Q1	1	1	1
Q2	1.34 (1.02, 1.76)	1.25 (0.94, 1.66)	1.06(0.77, 1.47)
Q3	3.13 (2.41, 4.05)	2.94 (2.23, 3.87)	1.43(1.02, 2.00)
P for trend	<0.001	<0.001	<0.001

Model 1 adjust for: none.

Model 2 adjust for: gender, age, race, education, marital, family PIR.

Model 3 adjust for: gender, age, race, education, marital, family PIR, smoking, drinking, BMI, Physical activate, HTN, CVD, HLD, Insulin use, Oral hypoglycemic use, DM, FPG, HbA1c.

### 5.3 Associations of AISI with uric acid levels and DKD

Table 3 presents the associations between UA levels and AISI, as well as its component indices, based on multivariable logistic regression models. After adjustment for all potential confounders, each 1 mg/dL increase in UA was associated with a 3.56 % rise in AISI (β = 0.0356, 95 % CI = 0.0103–0.0608, P = 0.0058). At higher UA levels, AISI increased by 7.01 %. In Model 3, UA levels showed significant positive correlations with neutrophil count (β = 0.0356, 95 % CI = 0.0103–0.0608, P = 0.0058) and monocyte count (β = 0.0140, 95 % CI = 0.0013–0.0266, P = 0.0303), but no significant associations with lymphocyte count (β = 0.0139, 95 % CI = −0.0276–0.0555, P = 0.5111) or platelet count (β = 0.0051, 95 % CI = −0.0044–0.0167, P = 0.2518). These findings suggest that elevated UA selectively activates myeloid immune cells, while exerting no direct effects on lymphocyte or platelet production. The strong association with neutrophils (P < 0.01) highlights them as the principal effector cells mediating the inflammatory effects of UA.

**Table 3:** The associations between uric acid levels and AISI.

Outcome	Level	Model	β value	95 % CI	P value
AISI(ln)	Continuous	Model1	0.0541	(0.0305, 0.0778)	<0.001
		Model2	0.0456	(0.0218, 0.0694)	<0.001
		Model3	0.0356	(0.0103, 0.0608)	0.0058
	Q1 (lowest)		1		
	Q2	Model1	−0.0143	(-0.1015, 0.0729)	0.7474
		Model2	−0.0190	(-0.1049, 0.0669)	0.6642
		Model3	−0.0101	(-0.0973, 0.0770)	0.8200
	Q3	Model1	0.1237	(0.0373, 0.2101)	0.0050
		Model2	0.0924	(0.0060, 0.1788)	0.0360
		Model3	0.0701	(-0.0202, 0.1604)	0.1279
	P for trend				0.0676
Abs_Neut	Continuous	Model1	0.0229	(0.0106, 0.0353)	<0.001
		Model2	0.0268	(0.0145, 0.0391)	<0.001
		Model3	0.0209	(0.0080, 0.0339)	0.0015
	Q1 (lowest)		1		
	Q2	Model1	−0.0072	(-0.0533, 0.0388)	0.7583
		Model2	0.0071	(-0.0387, 0.0529)	0.7601
		Model3	0.0169	(-0.0278, 0.0615)	0.4587
	Q3	Model1	0.0512	(0.0066, 0.0958)	0.0243
		Model2	0.0644	(0.0192, 0.1096)	0.0052
		Model3	0.0526	(0.0072, 0.0980)	0.0232
	P for trend				0.0206
Abs_Mono	Continuous	Model1	0.0360	(0.0255, 0.0465)	<0.001
		Model2	0.0269	(0.0162, 0.0376)	<0.001
		Model3	0.0225	(0.0109, 0.0341)	<0.001
	Q1 (lowest)		1		
	Q2	Model1	0.0550	(0.0134, 0.0966)	0.0096
		Model2	0.0390	(-0.0028, 0.0808)	0.0674
		Model3	0.0415	(-0.0009, 0.0839)	0.0553
	Q3	Model1	0.1207	(0.0796, 0.1619)	<0.001
		Model2	0.0861	(0.0439, 0.1283)	<0.001
		Model3	0.0757	(0.0314, 0.1200)	<0.001
	P for trend				<0.001
Abs_Lymph	Continuous	Model1	−0.0053	(-0.0169, 0.0062)	0.3636
		Model2	0.0135	(0.0017, 0.0253)	0.0244
		Model3	0.0140	(0.0013, 0.0266)	0.0303
	Q1 (lowest)		1		
	Q2	Model1	0.0187	(-0.0282, 0.0655)	0.4344
		Model2	0.0572	(0.0119, 0.1024)	0.0133
		Model3	0.0601	(0.0150, 0.1052)	0.0089
	Q3	Model1	0.0027	(-0.0406, 0.0460)	0.9031
		Model2	0.0717	(0.0277, 0.1157)	0.0014
		Model3	0.0721	(0.0264, 0.1177)	0.0020
	P for trend				0.0047
Platelets	Continuous	Model1	−0.0101	(-0.0201, −0.0002)	0.0462
		Model2	0.0054	(-0.0046, 0.0154)	0.2889
		Model3	0.0061	(-0.0044, 0.0167)	0.2518
	Q1 (lowest)		1		
	Q2	Model1	−0.0434	(-0.0844, −0.0023)	0.0383
		Model2	−0.0080	(-0.0487, 0.0328)	0.7016
		Model3	−0.0084	(-0.0499, 0.0332)	0.6938
	Q3	Model1	−0.0456	(-0.0850, −0.0062)	0.0234
		Model2	0.0136	(-0.0269, 0.0541)	0.5104
		Model3	0.0139	(-0.0276, 0.0555)	0.5111
	P for trend				0.3654

Model 1 adjust for: none.

Model 2 adjust for: gender, age, race, education, marital, family PIR.

Model 3 adjust for: gender, age, race, education, marital, family PIR, smoking, drinking, BMI, Physical activate, HTN, CVD, HLD, Insulin use, Oral hypoglycemic use, DM, FPG, HbA1c.

Table 4 summarizes the logistic regression models assessing the associations between DKD prevalence and inflammatory indices. We standardized AISI by natural logarithmic transformation and expressed the risk per one standard deviation (SD) increase in ln(AISI). Even after full adjustment for confounding factors, each 1-SD increase in ln(AISI) was associated with a 25 % higher risk of DKD (OR = 1.25, 95 % CI = 1.10–1.42), demonstrating a significant positive correlation. Both elevated AISI and neutrophil count (OR = 1.17, 95 % CI = 1.03–1.33) were identified as independent risk factors for DKD, whereas higher lymphocyte count appeared to confer a protective effect (OR = 0.83, 95 % CI = 0.73–0.95).

**Table 4:** The associations between DKD and AISI.

	OR (95 % CI)		
	Model1	Model2	Model3
DKD			
AISI	1.40 (1.26, 1.54)	1.36 (1.21, 1.52)	1.25 (1.10, 1.42)
Neutrophil	1.34 (1.21, 1.48)	1.34 (1.20, 1.50)	1.17 (1.03, 1.33)
Monocyte	1.27 (1.15, 1.40)	1.13 (1.01, 1.26)	1.03 (0.91, 1.16)
Lymphocyte	0.80 (0.72, 0.88)	0.87 (0.78, 0.97)	0.83 (0.73, 0.95)
Platelets	0.88 (0.80, 0.97)	1.03 (0.93, 1.15)	1.09 (0.96, 1.24)

Model 1 adjust for: none.

Model 2 adjust for: gender, age, race, education, marital, family PIR.

Model 3 adjust for: gender, age, race, education, marital, family PIR, smoking, drinking, BMI, Physical activate, HTN, CVD, HLD, Insulin use, Oral hypoglycemic use, DM, FPG, HbA1c.

### 5.4 Mediating role of AISI

**Fig. 2:**
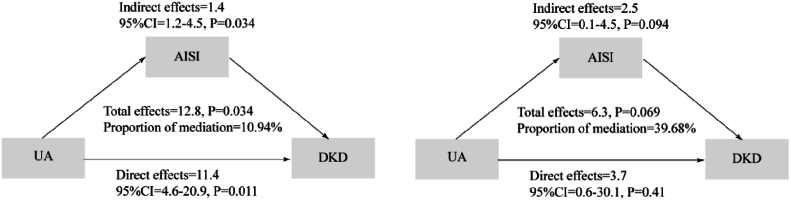
Analysis of the association between UA and DKD mediated by AISI. Exploratory subgroup analysis in participants with hyperuricemia (UA ≥7 mg/dL in men, ≥6 mg/dL in women); the total effect was borderline significant (p = 0.069).

Fig. 2 illustrates the mediation analysis results. In the overall population, AISI mediated 10.94 % of the association between UA and DKD. Notably, among participants with hyperuricemia, the proportion mediated increased substantially to 39.68 %.

### 5.5 Model construction and evaluation

**Fig. 3:**
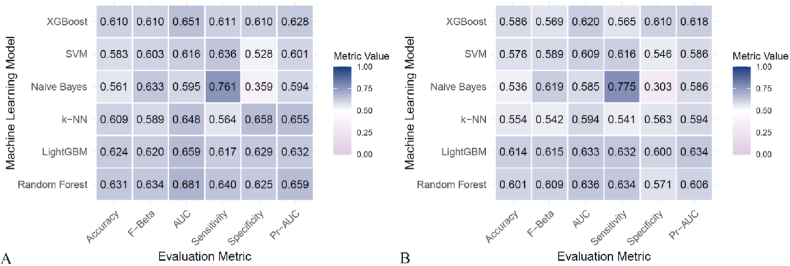
Heatmaps of six machine learning models (Random Forest, LightGBM, K-NN, Naive Bayes, SVM, XGBoost) for DKD prediction. Performance was assessed by Accuracy, F-Beta, ROC AUC, PR AUC, Sensitivity, and Specificity. (A) Training set: Random Forest ranked highest, Naive Bayes lowest. (B) Validation set: Random Forest again showed best overall performance.

**Fig. 4:**
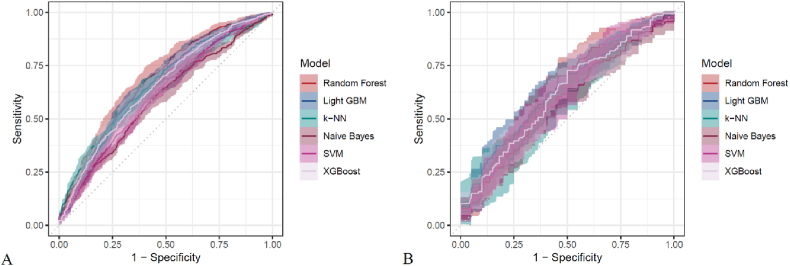
ROC curves of six machine learning models (Random Forest, LightGBM, K-NN, Naive Bayes, SVM, XGBoost) for DKD prediction in the (A) training and (B) validation sets. Random Forest consistently showed the highest AUC, confirming its superior discriminative ability.

**Fig. 5:**
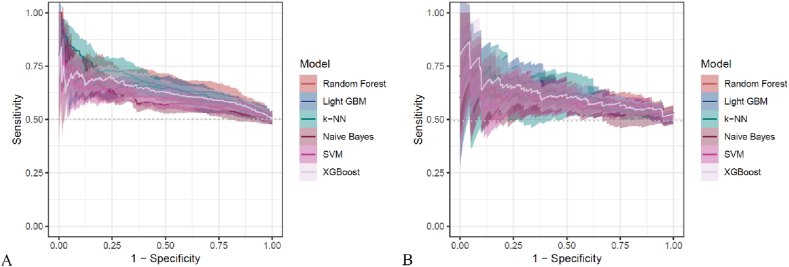
Precision–recall curves of six machine learning models (Random Forest, LightGBM, K-NN, Naive Bayes, SVM, XGBoost) for DKD prediction in the (A) training and (B) validation sets. Random Forest achieved the highest PR AUC, confirming its superior performance across imbalanced outcomes.

Fig. 3 presents heatmaps of the six models (Random Forest, LightGBM, K-KNN, Naive Bayes, SVM, XGBoost) trained and validated. Their performance was evaluated using ROC AUC (Fig. 4, Fig. S1B), PR AUC (Fig. 5, Fig. S1C), Accuracy (Fig. S1A), F-Beta (Fig. S1D), Sensitivity (Fig. S1E), and Specificity (Fig. S1F). Additional models incorporating demographic variables, AISI, and its components were also constructed (Table 5, Table 6).In the training set, Random Forest achieved the best overall performance, with Accuracy (0.631), F-Beta (0.634), ROC AUC (0.681), Sensitivity (0.640), Specificity (0.625), and PR AUC (0.659) ranking highest. XGBoost and LightGBM followed, with ROC AUCs of 0.651 and 0.659, PR AUCs of 0.628 and 0.632, Accuracy of 0.610 and 0.624, and F-Beta of 0.610 and 0.620, respectively. K-KNN and SVM demonstrated moderate performance, while Naive Bayes performed worst, particularly in ROC AUC (0.595) and PR AUC (0.594) (Table 5). In the validation set, Random Forest again outperformed the others, with Accuracy (0.601), F-Beta (0.609), ROC AUC (0.636), Sensitivity (0.634), Specificity (0.571), and PR AUC (0.606), achieving the best overall results (Table 6). These findings confirm Random Forest as the most effective model for predicting DKD risk. The calibration curve indicated that the Random Forest model demonstrated good calibration performance (Fig. S2).

**Table 5:** Performance of six machine learning models for DKD prediction in the training set.

Model	Accuracy	F Beta	Area under the ROC curve	Sensitivity	Specificity	Area under the PR curve
Random Forest	0.631	0.634	0.681	0.640	0.625	0.659
Light GBM	0.624	0.620	0.659	0.617	0.629	0.632
K-KNN	0.609	0.589	0.648	0.564	0.658	0.655
Naive Bayes	0.561	0.633	0.595	0.761	0.359	0.594
SVM	0.583	0.603	0.616	0.636	0.528	0.601
XGBoost	0.610	0.610	0.651	0.611	0.610	0.628
P	<.001^a^	<.001^a^	<.001^b^	<.001^a^	<.001^a^	<.001^a^

a ANOVA test.

b Kruskal-Wallis.

**Table 6:** Performance of six machine learning models for DKD prediction in the validation set.

Model	Accuracy	F Beta	Area under the ROC curve	Sensitivity	Specificity	Area under the PR curve
Random Forest	0.601	0.609	0.636	0.634	0.571	0.606
Light GBM	0.614	0.615	0.633	0.632	0.600	0.634
K-KNN	0.554	0.542	0.594	0.541	0.563	0.594
Naive Bayes	0.536	0.619	0.585	0.775	0.303	0.586
SVM	0.576	0.589	0.609	0.616	0.546	0.586
XGBoost	0.586	0.569	0.620	0.565	0.610	0.618
P	<.001^a^	<.001^a^	<.001^b^	<.001^a^	<.001^a^	<.001^a^

a ANOVA test.

b Kruskal-Wallis.

### 5.6 Interpretation of AISI and its components in DKD risk prediction using SHAP and LIME

**Fig. 6:**
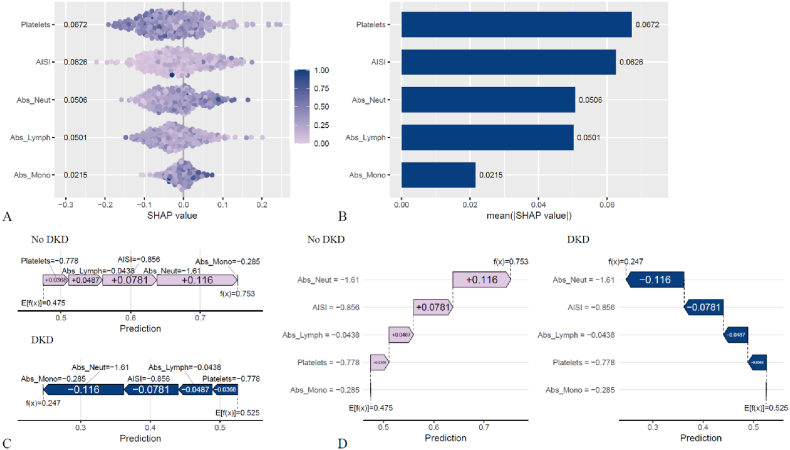
SHAP analysis of the Random Forest model for DKD prediction. (A) SHAP summary plot showing contributions of AISI and its components. (B) Mean SHAP values ranking feature importance. (C, D) Force and waterfall plots illustrating case-level predictions, where lighter-colored features indicate stronger contributions.

SHAP analysis in the training set quantified the contributions of AISI and its components to DKD risk (Fig. 6A and B). Positive contributors included AISI (0.0626), Abs_Neut (0.0506), and Abs_Mono (0.0215). Negative contributors included Platelets (0.0672) and Abs_Lymph (0.0501). Force and waterfall plots (Fig. 6C and D) provided case-level explanations for the Random Forest model. Lighter-colored features indicated stronger contributions toward outcome prediction. For participants predicted as DKD-free, the baseline probability was 0.475, which increased to 0.753 after incorporating feature effects.LIME was further applied for individualized interpretation. For an estimated 0.753 probability of being DKD-free, the ranges −0.86 < AISI ≤ −0.601, −1.61 < Abs_Neut ≤ −0.6893, and −0.153 < Abs_Lymph ≤0.504 were identified as contributing factors (Fig. S3).

#### 5.6.1 Sensitivity analysis

To validate the stability of the machine learning models, we first trained the models using the dataset without SMOTE processing. In the training set, Random Forest again achieved the best overall performance, with leading values for accuracy (0.618), F-beta (0.630), AUC-ROC (0.668), sensitivity (0.646), specificity (0.592), and AUC-PR (0.654) (Table S1). In the validation set, Random Forest also demonstrated the strongest performance, with accuracy (0.575), F-beta (0.565), AUC-ROC (0.635), sensitivity (0.583), specificity (0.574), and AUC-PR (0.605), achieving the highest overall predictive capability (Table S2, Fig. S4). The Random Forest model remained the best-performing algorithm both with and without SMOTE, and the difference in AUC-ROC between the two conditions was less than 0.02, further supporting the robustness of the findings.

To further assess the stability of the results, multiple imputation was applied to handle missing values, and the association between UA and DKD was re-evaluated. After adjusting for potential confounding variables, UA remained positively associated with DKD (OR = 1.15, 95 % CI = 1.04–1.27). The association observed after multiple imputation was consistent with that obtained before imputation (Table S3).

Finally, the dataset from 2011 to 2018 was used as an external test set. Random Forest again achieved the best performance, with an AUC-ROC of 0.672 and strong results across other evaluation metrics, demonstrating the highest overall predictive performance (Table S4).

## 6 Discussion

The present cross-sectional study, based on nationally representative NHANES 1999–2018 data, confirmed a positive association between serum uric acid and diabetic kidney disease after extensive multivariable adjustment. Even after applying survey-weighted regression, each 1 mg/dL increase in serum uric acid remained associated with a 14 % higher odds of diabetic kidney disease (adjusted OR = 1.14, 95 % CI 1.04–1.26), consistent with previous epidemiological evidence [24,25].

Systemic inflammation, as reflected by the AISI, partially mediated this association, with an indirect effect proportion of 10.94 % in the overall population. The relatively low mediated proportion indicates that inflammation is only one of several pathways linking uric acid to diabetic kidney disease; direct crystal-independent mechanisms, oxidative stress, RAAS activation, endothelial dysfunction, and hemodynamic changes likely remain dominant contributors [26]. In an exploratory subgroup analysis restricted to participants with hyperuricemia, the mediated proportion increased to 39.68 %; however, the total effect in this subgroup was only borderline significant (p = 0.069), limiting interpretability.

Unlike previous studies that primarily focused on single inflammatory indicators such as the neutrophil-to-lymphocyte ratio (NLR) or the systemic immune-inflammation index (SII), the present study is the first to introduce AISI into the UA–DKD research framework to quantify the contribution of UA-induced systemic inflammatory imbalance to kidney disease. By integrating neutrophil, monocyte, platelet, and lymphocyte counts, AISI not only reflects heightened pro-inflammatory activity of myeloid cells but also implies potential impairment of lymphocyte-mediated immune regulation, thereby aptly capturing the “pro-inflammatory versus anti-inflammatory” disequilibrium triggered by uric acid [27,28].

Existing basic research indicates that UA can perturb the inflammatory network through multiple pathways. First, UA activates the NLRP3 inflammasome axis, leading to increased numbers of monocytes and macrophages. Monosodium urate (MSU) crystals initially stimulate Toll-like receptors (TLRs) on monocyte/macrophage surfaces, providing a “first signal” via the TLR–NF-κB pathway, followed by a “second signal” triggered by MSU crystals themselves, potassium efflux, or lysosomal rupture, which activates the NLRP3–caspase-1 cascade and promotes IL-1β/IL-18 release. Complement component C5a further amplifies this pathway and the expression of pro–IL-1β; moreover, soluble UA can also activate NLRP3 under hypoxic or metabolic stress conditions, resulting in enhanced recruitment and activation of monocytes [[28], [29], [30], [31], [32]].Second, IL-1β and other chemokines drive rapid neutrophil recruitment [33,34]. MSU-induced IL-1β release promotes massive neutrophil infiltration, while MSU directly induces neutrophil extracellular trap (NET) formation (NETosis). NETs exacerbate acute inflammatory responses yet also participate in self-limiting clearance processes, leaving neutrophils in a persistently hyperactivated state [35,36].Third, platelets become key accomplices of inflammation in hyperuricemic conditions. UA damages vascular endothelium, causing endothelial dysfunction, reduced nitric oxide bioavailability, externalization of phosphatidylserine, and microparticle release [37,38]. These changes strongly activate platelets, fostering the formation of various platelet–leukocyte aggregates (with neutrophils, monocytes, and others), which promote intravascular microthrombosis, thereby aggravating both inflammation and vascular occlusion [39]. In turn, inflammation itself promotes thrombopoiesis, increasing platelet numbers [40].Fourth, hyperuricemia-induced inflammation disrupts adaptive immunity, shifting the Th17/Treg balance toward a pro-inflammatory state. Clinically, gout patients often exhibit reduced lymphocyte counts or functional suppression, alongside maladaptive training of monocytes and other innate immune cells, leading to impaired lymphocyte regulatory capacity [41,42]. Taken together, the formulation of AISI is biologically well aligned with the multifaceted perturbations induced by UA on the immune system. This explains why AISI serves as an ideal mediator, statistically capturing a significant portion of the effect of UA on DKD.

The random forest model incorporating AISI and its components achieved only moderate discriminative performance, which did not exceed that of conventional clinical risk scores relying predominantly on eGFR and albuminuria (reported AUC 0.65–0.80 in comparable NHANES-based diabetic cohorts). SHAP analysis nevertheless confirmed AISI and absolute neutrophil count as important positive contributors, supporting the biological relevance of inflammation even when overall predictive gain is limited.

AISI, introduced in 2018 as an innovative hematological metric, has been widely validated in linking systemic inflammatory responses with multiple diseases [43]. The present study confirms that AISI more accurately reflects the immunological effects of UA [33] and may serve as a pivotal inflammatory hub connecting “metabolic burden” with “end-organ damage.”. In this context, Huang et al. reported a J-shaped association between AISI and chronic kidney disease, with an inflection point at 181.27, but found a positive correlation in individuals with lower eGFR [44]. Furthermore, other studies have shown that once inflammatory biomarkers such as AISI exceed a certain threshold in patients with diabetes, risks of cardiovascular mortality (HR = 1.73) and cancer-related mortality (HR = 1.20) increase significantly [45,46]. Sun et al. also demonstrated a dose-dependent relationship between AISI and proteinuria, with each one-unit increase in log_2_AISI associated with a 31 % higher prevalence of proteinuria, along with interactions involving smoking, alcohol consumption, and blood pressure—findings consistent with our results [47]. Collectively, accumulating evidence highlights the strong association between AISI and multisystem diseases, underscoring its prognostic value.

Clinically, these findings carry important implications. As a simple and easily accessible hematological parameter, AISI holds promise for the early identification of diabetic patients with hyperuricemia who are at increased risk of kidney injury. If the relationship between UA and DKD is partly mediated by AISI, this suggests that reducing AISI levels may represent an effective entry point for interventions targeting the UA–DKD axis. This opens new therapeutic avenues: beyond conventional uric acid–lowering strategies, combining anti-inflammatory interventions—such as NLRP3 inhibitors, IL-1β blockers, or lifestyle modifications—may yield greater renoprotective benefits. Incorporating AISI into routine clinical assessment could therefore not only facilitate risk stratification but also provide a measurable target for disrupting the “metabolism–inflammation–renal injury” cascade.

## 7 Limitations

Of course, this study has several limitations. First, NHANES is a cross-sectional survey, which prevents us from establishing causal relationships. Second, although we adjusted for multiple potential confounders, residual confounding cannot be entirely ruled out. Third, while AISI reflects systemic inflammatory burden, it does not encompass cytokines or molecular-level inflammatory markers, which may lead to an underestimation of the true inflammatory effect. Nevertheless, our findings are the first to validate, through the lens of AISI, the hypothesis that UA accelerates the development of DKD by disrupting the inflammatory network. This provides important evidence to support future longitudinal studies and interventional strategies.

## 8 Conclusion

This study provides evidence that AISI mediates the positive association between UA and accelerated renal impairment in patients with diabetes, highlighting the significant mediating role of systemic inflammation in this relationship. These insights add to the growing body of evidence supporting the clinical utility of AISI and offer valuable perspectives for developing early intervention strategies in populations at risk of DKD.

## CRediT authorship contribution statement

**Jiaying Wang:** Writing – original draft. **Weijing Liu:** Writing – review & editing. **Jiaoyan Li:** Writing – review & editing. **Mengxiao Li:** Data curation. **Heyan Feng:** Formal analysis. **Shangfei Liu:** Formal analysis. **Yanzhe Cheng:** Data curation. **Wei Li:** Writing – review & editing.

## Ethics approval and consent to participate

The National Center for Health Statistics Ethics Review Board has approved the implementation of NHANES.

## Consent for publication

Not applicable.

## Funding

The author(s) declare that financial support was received for the research and publication of this article. This work was supported by the construction project of the Grassroots Inheritance 10.13039/100028250Studio for Characteristic Diseases (Syndromes) of Traditional Chinese Medicine in Heping Street Community, Chaoyang District, Beijing.

## Conflict of interest

The authors declare that the research was conducted in the absence of any commercial or financial relationships that could be construed as a potential conflict of interest.

## Data Availability

The datasets generated and analysis during the current study are available in the NHANES, www.cdc.gov/nchs/NHANEs/.
