# Supplementary material for: Cross-sectional mediation analysis of systemic inflammation in the association between serum uric acid and diabetic kidney disease: Evidence from NHANES 1999–2018
Source: Metabol Open. 2025 Dec 2;28:100426. doi: 10.1016/j.metop.2025.100426 (PMC12721052; doi:10.1016/j.metop.2025.100426)
Supplement: Multimedia component 2 [file mmc2.docx]

**Cross-Sectional Mediation Analysis of Systemic Inflammation in the Association Between Serum Uric Acid and Diabetic Kidney Disease: Evidence from NHANES 1999–2018**

Jiaying Wang, Wei Li*, Weijing Liu, Jiaoyan Li, Mengxiao Li, Heyan Feng, Shangfei Liu, Yanzhe Cheng

Corresponding author : Wei Li

E-mail: [17334371326@163.com](mailto:17334371326@163.com)


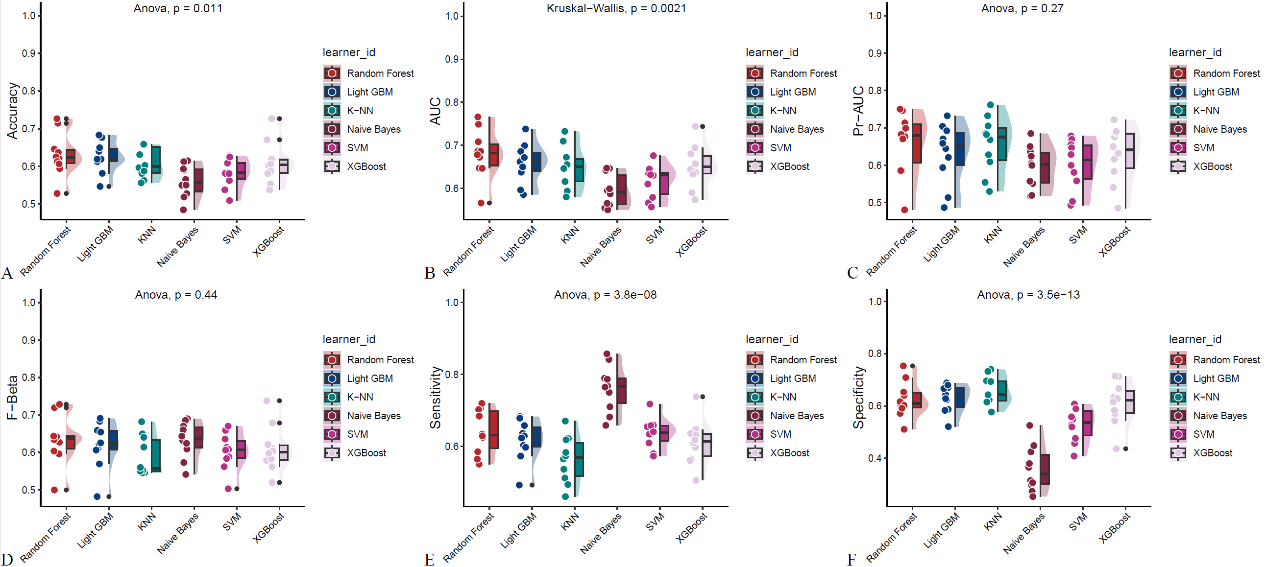
**Fig S1. Comparative performance of six machine learning models—Random Forest, LightGBM, K-NN, Naive Bayes, SVM, and XGBoost—for predicting diabetic kidney disease (DKD).** Each violin/box plot shows the distribution of model performance across resampling iterations. Evaluation metrics include (A) Accuracy, reflecting overall correctness; (B) ROC AUC, assessing global discriminative ability; (C) PR AUC, evaluating performance under class imbalance; (D) F-Beta score, balancing precision and recall; (E) Sensitivity, measuring true positive rate; and (F) Specificity, measuring true negative rate. Statistical significance of performance differences was assessed using ANOVA or Kruskal–Wallis tests, with P values displayed above each panel. Random Forest consistently outperformed other models across most metrics, particularly in ROC AUC, PR AUC, Sensitivity, and Specificity, while Naive Bayes showed the weakest performance.


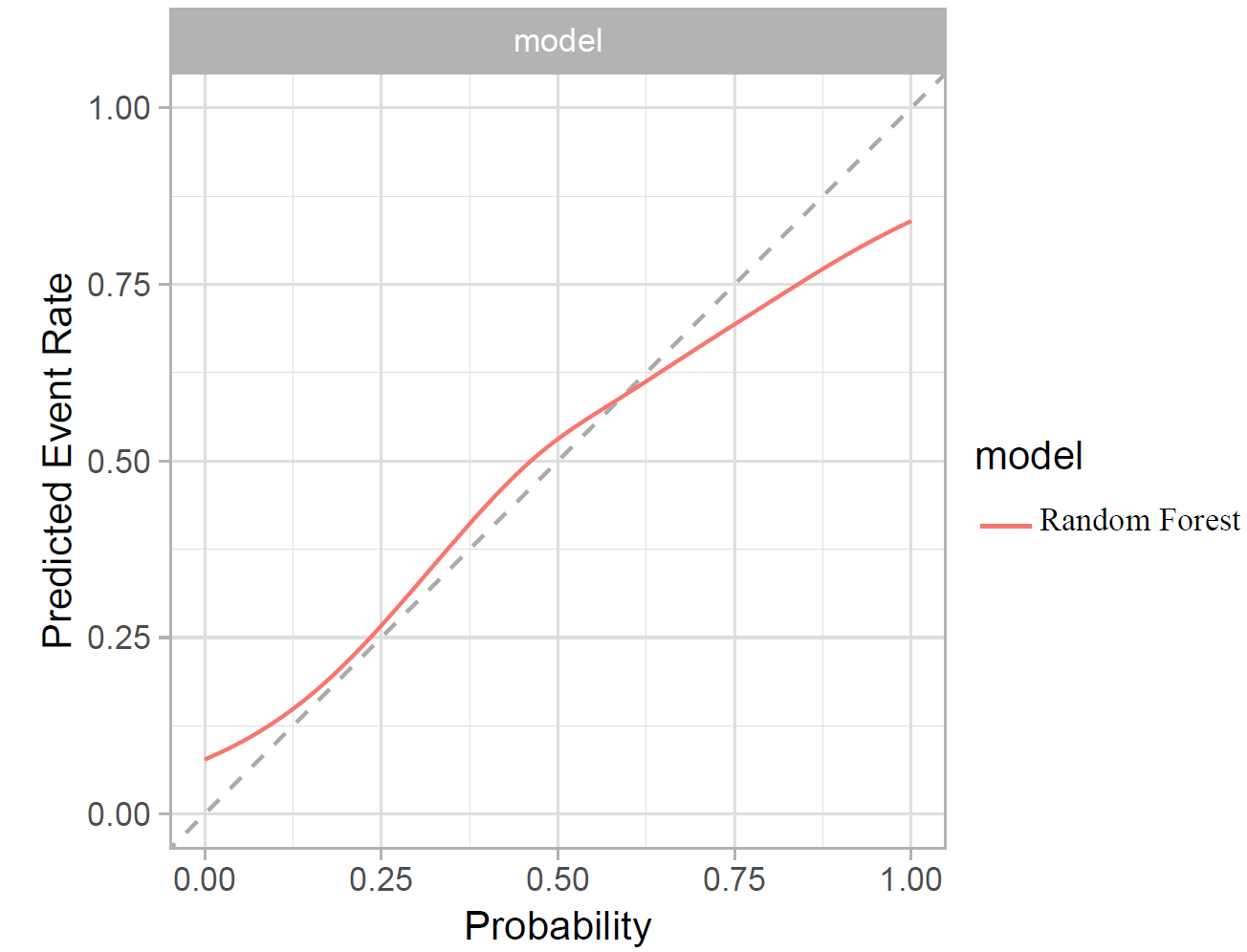


**Fig.S2. Calibration plot of the Random Forest model for predicting DKD risk.** The calibration curve evaluates the agreement between predicted probabilities from the Random Forest model and observed actual probabilities of DKD across deciles of risk. The x-axis represents the mean predicted probability of DKD within each decile, while the y-axis shows the corresponding observed proportion of DKD cases (empirical frequency). The diagonal dashed line (y = x) indicates perfect calibration, where predicted risks exactly match observed outcomes. The solid blue line depicts the model’s calibration performance; its close alignment with the ideal reference line demonstrates good overall calibration, indicating that the model’s predicted probabilities are well-calibrated and reliable across the full risk spectrum. The Hosmer–Lemeshow goodness-of-fit test yielded a non-significant p-value (p = 0.32), further supporting the absence of substantial miscalibration. Shaded areas or error bars (if present) represent 95% confidence intervals around the observed proportions. This plot was generated using 10-fold cross-validation on the validation set to ensure robustness against overfitting.


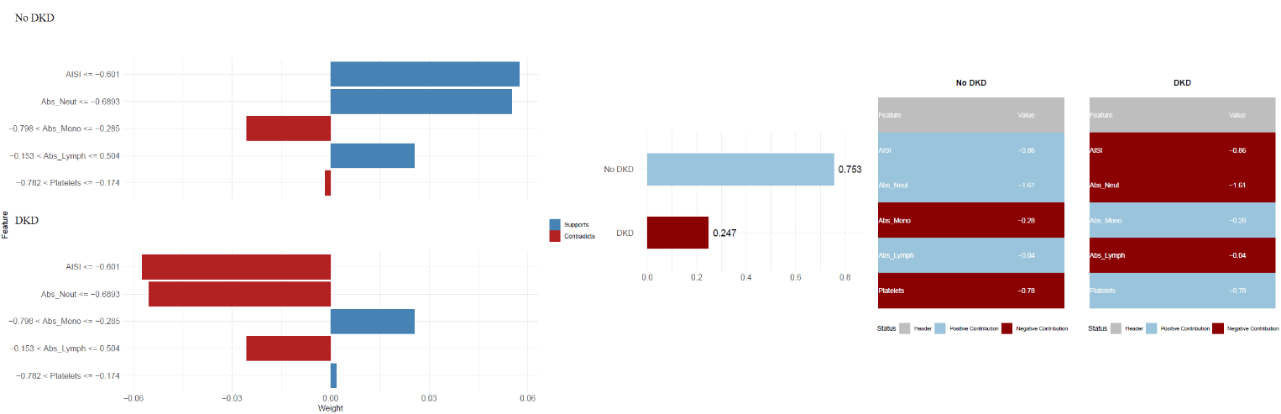


**Fig.S3. LIME interpretation of AISI and its components for DKD prediction.** (A) Feature weights for participants predicted as DKD-free and DKD. (B) Baseline probability of being DKD-free (0.475) increased to 0.753 after feature effects. (C) Feature ranges identified by LIME as supporting or contradicting DKD outcomes.

**Table S1. Model performance in training set without SMOTE**

| **Model** | **Accuracy** | **F Beta** | **Area under the ROC curve** | **Sensitivity** | **Specificity** | **Area under the PR curve** |
| --- | --- | --- | --- | --- | --- | --- |
| Random Forest | 0.618 | 0.630 | 0.668 | 0.646 | 0.592 | 0.654 |
| Light GBM | 0.605 | 0.616 | 0.655 | 0.629 | 0.581 | 0.638 |
| K-KNN | 0.590 | 0.584 | 0.622 | 0.572 | 0.608 | 0.624 |
| Naive Bayes | 0.562 | 0.634 | 0.594 | 0.753 | 0.368 | 0.603 |
| SVM | 0.577 | 0.606 | 0.616 | 0.645 | 0.514 | 0.610 |
| XGBoost | 0.590 | 0.598 | 0.646 | 0.607 | 0.575 | 0.629 |
| P | <.001^a^ | <.001^a^ | <.001^b^ | <.001^a^ | <.001^a^ | <.001^a^ |
| ^a^ANOVA test; ^b^Kruskal-Wallis  **Table S2. Model performance in validation set without SMOTE** | | | | | | |
| **Model** | **Accuracy** | **F Beta** | **Area under the ROC curve** | **Sensitivity** | **Specificity** | **Area under the PR curve** |
| Random Forest | 0.575 | 0.565 | 0.635 | 0.583 | 0.574 | 0.605 |
| Light GBM | 0.608 | 0.587 | 0.645 | 0.584 | 0.634 | 0.626 |
| K-KNN | 0.566 | 0.518 | 0.583 | 0.486 | 0.645 | 0.583 |
| Naive Bayes | 0.601 | 0.625 | 0.622 | 0.704 | 0.507 | 0.590 |
| SVM | 0.588 | 0.565 | 0.618 | 0.569 | 0.614 | 0.572 |
| XGBoost | 0.600 | 0.580 | 0.647 | 0.577 | 0.625 | 0.630 |
| P | <.001^a^ | <.001^a^ | <.001^b^ | <.001^a^ | <.001^a^ | <.001^a^ |
| ^a^ANOVA test; ^b^Kruskal-Wallis | | | | | | |

**Table S3. External validation on NHANES 2011–2018**

| DKD | OR (95% CI) | | |
| --- | --- | --- | --- |
|  | Model1 | Model2 | Model3 |
| Continuous uric acid | | | |
| uric acids | 1.37 (1.47,1.57) | 1.45 (1.25, 1.67) | 1.15(1.04, 1.27) |
| Uric acid categories | | | |
| Q1 | 1 | 1 | 1 |
| Q2 | 1.24 (1.12, 1.66) | 1.15 (0.92, 1.56) | 1.05(0.76, 1.45) |
| Q3 | 3.10 (2.21, 4.15) | 2.84 (2.13, 3.77) | 1.44(1.01, 2.20) |
| P for trend | <0.001 | <0.001 | <0.001 |

Model 1 adjust for: none

Model 2 adjust for: gender, age, race, education, marital, family PIR,

Model 3 adjust for: gender, age, race, education, marital, family PIR, smoking, drinking, BMI, Physical activate, HTN, CVD, HLD, Insulin use, Oral hypoglycemic use, DM, FPG , HbA1c.

**Table S4. Association between uric acid and DKD adjusted for urate-lowering therapy**

| **Model** | **Accuracy** | **F Beta** | **Area under the ROC curve** | **Sensitivity** | **Specificity** | **Area under the PR curve** |
| --- | --- | --- | --- | --- | --- | --- |
| Random Forest | 0.638 | 0.652 | 0.672 | 0.672 | 0.603 | 0.668 |
| Light GBM | 0.627 | 0.632 | 0.672 | 0.638 | 0.613 | 0.664 |
| K-KNN | 0.565 | 0.570 | 0.594 | 0.573 | 0.561 | 0.599 |
| Naive Bayes | 0.545 | 0.633 | 0.594 | 0.777 | 0.310 | 0.605 |
| SVM | 0.581 | 0.608 | 0.621 | 0.645 | 0.520 | 0.618 |
| XGBoost | 0.613 | 0.621 | 0.655 | 0.629 | 0.592 | 0.650 |
| P | <.001^a^ | <.001^a^ | <.001^b^ | <.001^a^ | <.001^a^ | <.001^a^ |
| ^a^ANOVA test; ^b^Kruskal-Wallis | | | | | | |


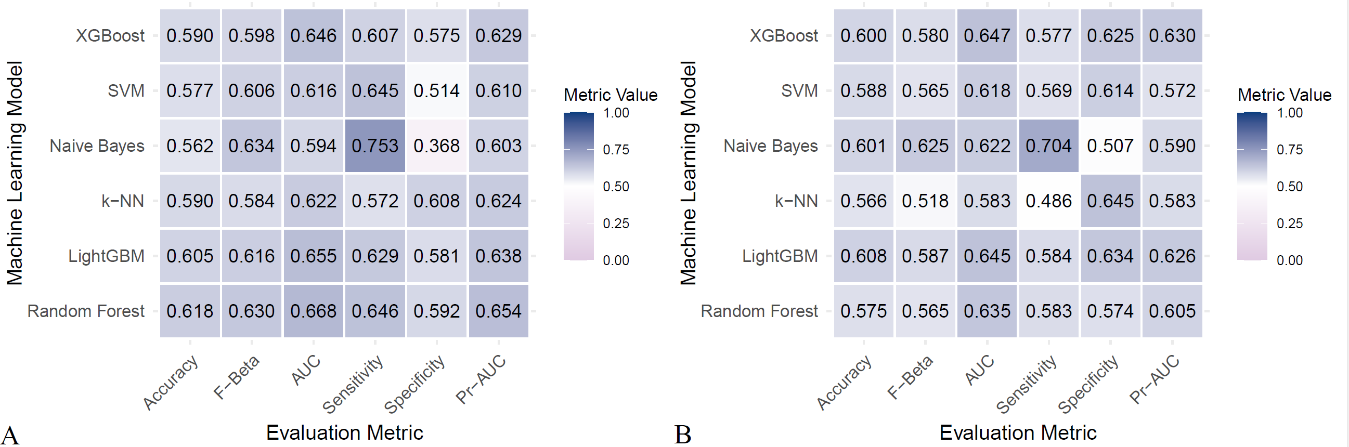


**Fig. S4. Performance comparison of six machine learning models for diabetic kidney disease prediction.** Bar plots display model performance metrics (Accuracy, F-Beta, ROC AUC, Sensitivity, Specificity, PR AUC) on the validation set without SMOTE oversampling. Random Forest achieved the highest scores in ROC AUC (0.635) and PR AUC (0.605), demonstrating robust predictive capability for DKD risk stratification.
