# Supplementary material for: Cross-sectional mediation analysis of systemic inflammation in the association between serum uric acid and diabetic kidney disease: Evidence from NHANES 1999–2018
Source: Metabol Open. 2025 Dec 2;28:100426. doi: 10.1016/j.metop.2025.100426 (PMC12721052; doi:10.1016/j.metop.2025.100426)
Supplement: Multimedia component 4 [file mmc4.docx]

| NHANES Original Variable Name | Analytical Variable / Clinical Meaning |
| --- | --- |
| SEQN | **Unique participant identifier** |
| RIDAGEYR | **Age (years)** |
| RIAGENDR | **Sex (1 = Male, 2 = Female)** |
| RIDRETH1 | **Race/ethnicity (1 = Mexican American, 2 = Other Hispanic, 3 = Non-Hispanic White, 4 = Non-Hispanic Black, 5 = Other Race – Including Multi-Racial)** |
| DMDMARTL | **Marital status** |
| DMDEDUC2 | **Education level (years of schooling)** |
| INDFMPIR | **Family income-to-poverty ratio (PIR)** |
| BMXBMI | **Body mass index (kg/m²)** |
| LBXSCR | **Serum creatinine (mg/dL)** |
| Scr_umolL | **Serum creatinine (μmol/L; converted from LBXSCR × 88.4)** |
| LBXSBUSI | **Blood urea nitrogen (BUN, mg/dL)** |
| BUN_mmolL | **Blood urea nitrogen (mmol/L; converted from LBXSBUSI × 0.357)** |
| LBXSUA | **Serum uric acid (mg/dL)** |
| URXUMA | **Urinary albumin (μg/mL)** |
| URXUCR | **Urinary creatinine (mg/dL)** |
| ACR | **Urine albumin-to-creatinine ratio (mg/g; calculated as 100 × URXUMA / URXUCR)** |
| eGFR | **Estimated glomerular filtration rate (mL/min/1.73m²), calculated using the CKD-EPI 2009 equation** |
| LBXGH | **Glycated hemoglobin (HbA1c, %)** |
| LBXGLU | **Fasting plasma glucose (mg/dL)** |
| FPG_mmolL | **Fasting plasma glucose (mmol/L; converted from LBXGLU ÷ 18.0)** |
| DIQ010 | **Self-reported physician diagnosis of diabetes (1 = Yes, 2 = No)** |
| DID040 | **Age at first diagnosis of diabetes (years)** |
| DIQ050 | **Current insulin use (1 = Yes, 2 = No)** |
| DIQ070 | **Current oral hypoglycemic agent use (1 = Yes, 2 = No)** |
| anti_diabetic_med | **Any anti-diabetic medication use (derived from RXQ_RX drug names via text mining)** |
| BPXSY1–BPXSY4 | **Systolic blood pressure measurements (up to 4 readings, mmHg)** |
| BPXDI1–BPXDI4 | **Diastolic blood pressure measurements (up to 4 readings, mmHg)** |
| BPQ020 | **Self-reported physician diagnosis of hypertension (1 = Yes, 2 = No)** |
| BPQ040A | **Current antihypertensive medication use (1 = Yes, 2 = No)** |
| anti_htn_med | **Antihypertensive medication use (text-mined from RXQ_RX)** |
| LBXTC | **Total cholesterol (mg/dL)** |
| LBDLDL | **Low-density lipoprotein cholesterol (LDL-C, mg/dL)** |
| LBXTR | **Triglycerides (mg/dL)** |
| LBDHDD | **High-density lipoprotein cholesterol (HDL-C, mg/dL)** |
| anti_hld_med | **Lipid-lowering medication use (e.g., statins; text-mined from RXQ_RX)** |
| MCQ160B | **History of myocardial infarction (1 = Yes, 2 = No)** |
| MCQ160C | **History of stroke (1 = Yes, 2 = No)** |
| MCQ160E | **History of heart failure (1 = Yes, 2 = No)** |
| MCQ160N / MCQ160n | **Physician-diagnosed gout (1 = Yes, 2 = No)** |
| gout_ult_med | **Urate-lowering therapy use (e.g., allopurinol, febuxostat; text-mined from RXQ_RX)** |
| gout_colch_med | **Colchicine use (text-mined from RXQ_RX)** |
| SMQ020 | **Ever smoked ≥100 cigarettes in lifetime (1 = Yes, 2 = No)** |
| SMQ040 | **Current smoking status (1 = Every day, 2 = Some days, 3 = Not at all)** |
| ALCOHOL | **Consumed ≥12 alcoholic drinks in past year (derived from ALQ101/ALQ111)** |
| PA_cat | **Physical activity category (Inactive, Insufficient, Active, Highly active)** |
| MVPA_min_week | **Moderate-to-vigorous physical activity (minutes per week)** |
| LBXWBCSI | **White blood cell count (10⁹/L)** |
| LBXLYMNO / LBXLYPCT | **Absolute lymphocyte count (10⁹/L) or percentage (%)** |
| LBXNENO / LBXNEPCT | **Absolute neutrophil count (10⁹/L) or percentage (%)** |
| LBXMONO / LBXMOPCT | **Absolute monocyte count (10⁹/L) or percentage (%)** |
| LBXPLTSI | **Platelet count (10⁹/L)** |
| Abs_Lymph | **Derived absolute lymphocyte count (from % × WBC if absolute not available)** |
| Abs_Neut | **Derived absolute neutrophil count** |
| Abs_Mono | **Derived absolute monocyte count** |
| AISI | **Atherogenic Index of Systemic Inflammation = (Neutrophil × Monocyte × Platelet) / Lymphocyte** |
| RIDEXPRG | **Pregnant at time of examination (1 = Yes)** |
